# LncRNA ADAMTS9-AS2 inhibits cell proliferation and decreases chemoresistance in clear cell renal cell carcinoma via the miR-27a-3p/FOXO1 axis

**DOI:** 10.18632/aging.102154

**Published:** 2019-08-10

**Authors:** Er-lin Song, Li Xing, Liang Wang, Wen-ting Song, Dan-bin Li, Yi Wang, Yi-wei Gu, Ming-ming Liu, Wen-jun Ni, Peng Zhang, Xin Ma, Xu Zhang, Jie Yao, Yang Chen, Rui-hua An

**Affiliations:** 1 Department of Urinary Surgery, The First Affiliated Hospital of Harbin Medical University, Harbin 150007, Heilongjiang Province, P. R. China; 2 Department of Nephrology, The First Affiliated Hospital of Harbin Medical University, Harbin 150007, Heilongjiang Province, P. R. China; 3 Medical Department, The First Affiliated Hospital of Harbin Medical University, Harbin 150007, Heilongjiang Province, P. R. China; 4 Heilongjiang Academy of Medical Sciences, Harbin Medical University, Harbin 150081, Heilongjiang Province, P. R. China; 5 Department of Endocrinology, The First Affiliated Hospital of Harbin Medical University, Harbin 150007, Heilongjiang Province, P. R. China; 6 Department of Urology, Chinese PLA General Hospital/Chinese PLA Medical Academy, Beijing 100036, P.R. China; 7 Department of Urological Surgery, Zhongnan Hospital of Wuhan University, Wuhan 430071, Hubei Province, P.R. China; 8 Department of Hematology and Medical Oncology, Beijing ChuiYangLiu Hospital, Beijing 100022, P. R. China

**Keywords:** ADAMTS9-AS2, miR-27a-3p, renal cell carcinoma, chemoresistance, proliferation, FOXO1

## Abstract

Accumulating evidence reveals the principal role of long noncoding RNAs in the progression of clear cell renal cell carcinoma (ccRCC). However, little is known about the underlying mechanism of ADAM metallopeptidase with thrombospondin type 1 motif, 9 antisense RNA 2 (ADAMTS9-AS2) in ccRCC. Here, bioinformatics analyses verified ADAMTS9-AS2 is a long noncoding RNA and its high expression was associated with better prognosis of ccRCC. ADAMTS9-AS2 was clearly downregulated in ccRCC clinical samples and cell lines. Clinical data showed low-expressed ADAMTS9-AS2 was correlated with worse overall survival in ccRCC patients. Next, miR-27a-3p was identified as an inhibitory target of ADAMTS9-AS2 by dual-luciferase reporter and RNA immunoprecipitation assays. Both overexpressed ADAMTS9-AS2 and underexpressed miR-27a-3p in ccRCC cell lines led to the inhibition of cell proliferation and the reduction of chemoresistance. Additionally, Forkhead Box Protein O1 (FOXO1) was confirmed as the inhibitory target of miR-27a-3p. Induced by ADAMTS9-AS2 overexpression, cell proliferation and chemoresistance exhibited an obvious reduction, FOXO1 expression showed an evident increase, but all were reversed after miR-27a-3p was simultaneously overexpressed. Collectively, these results suggest ADAMTS9-AS2 inhibits the progression and impairs the chemoresistance of ccRCC via miR-27a-3p-mediated regulation of FOXO1 and may serve as a prognostic biomarker and therapeutic target for ccRCC.

## INTRODUCTION

Renal cell carcinoma (RCC) is the second most common contributor to mortality in patients with urologic tumors and accounts for 2% of adult malignancies [1]. Globally, approximately 270 000 cases of kidney cancer are diagnosed each year, and 116 000 people die from this disease. Clear cell RCC (ccRCC) comprises approximately 90% of the histological subtypes [2], and thus, it is one of the most lethal urological malignancies. Although the treatment of ccRCC has been significantly improved in the last two decades, various limitations of diagnosis and treatment of ccRCC persist. Generally, the diagnosis of ccRCC primarily depends on computed tomography scans and magnetic resonance imaging, and ccRCC is resistant to chemotherapy and radiotherapy, especially advanced or metastatic ccRCC. Additionally, the target drugs for ccRCC treatment have inherent limitations, including incompatibility for certain patients [3] and unstable drug efficacy biomarkers [4]. Advanced studies are necessary to clarify the pathogenesis of ccRCC and further develop targeted therapeutic approaches for treating ccRCC.

Long noncoding RNAs (lncRNAs), which contain more than 200 nucleotides, represent one type of noncoding RNA [5]. Recently, increasing numbers of lncRNAs have been reported to be associated with various types of cancers, such as breast cancer, gastric cancer, colorectal cancer, lung cancer, and ovarian cancer [6–10]. Particularly in ccRCC, it has been reported that many related lncRNAs are involved in carcinogenesis and progression, such as TUG1 [11], HEIRCC [12], and CRNDE [13]. Despite the identification of numerous lncRNAs that are associated with ccRCC, the majority of lncRNAs remain unexplored. Hence, more attention should be given to the functional role of lncRNAs in ccRCC, which spurred us to conduct this study.

Notably, a common type of cancer-related lncRNA is the antisense partner of a protein-coding gene, such as HNF1A-AS1 [14] and GAS6-AS1 [15]. As previously reported, ADAM metallopeptidase with thrombospondin type 1 motif, 9 (ADAMTS9) antisense RNA 2 (ADAMTS9-AS2) has been identified as a novel tumor suppressor, which may inhibit the proliferation and migration of non-small cell lung cancer cells (NSCLC) [16]. LncRNA ADAMTS9-AS2 is an antisense transcript of the protein-coding gene ADAMTS9. This lncRNA/mRNA gene pair is located at chromosome 3p14.1, which is a region known to be absent in hereditary renal cancers [17]. To our knowledge, no studies have considered the functional role and concrete mechanisms of ADAMTS9-AS2 in ccRCC.

In the present work, we first analyzed the protein-coding potential, expression and survival of ADAMTS9-AS2 according to bioinformatics. Then, we investigated the expression pattern of ADAMTS9-AS2 in ccRCC tissues and cell lines, and we analyzed the correlation between ADAMTS9-AS2 expression and the clinicopathological characteristics of ccRCC patients. Using several different assays and software programs, the binding of microRNA-27a-3p (miR-27a-3p) with ADAMTS9-AS2 and the targeting of Forkhead Box Protein O1 (FOXO1) by miR-27a-3p were predicted and verified. Additionally, the functional role of ADAMTS9-AS2 and miR-27a-3p in cell proliferation and chemoresistance was analyzed. Finally, the mechanism through which ADAMTS9-AS2 affected cell proliferation and chemoresistance was explored. Our results indicated that ADAMTS9-AS2 inhibits cell proliferation and decreases chemotherapy resistance of ccRCC, and the molecular mechanism underlying this function is clarified.

## RESULTS

### Bioinformatics analyses of ADAMTS9-AS2

**Figure 1:**
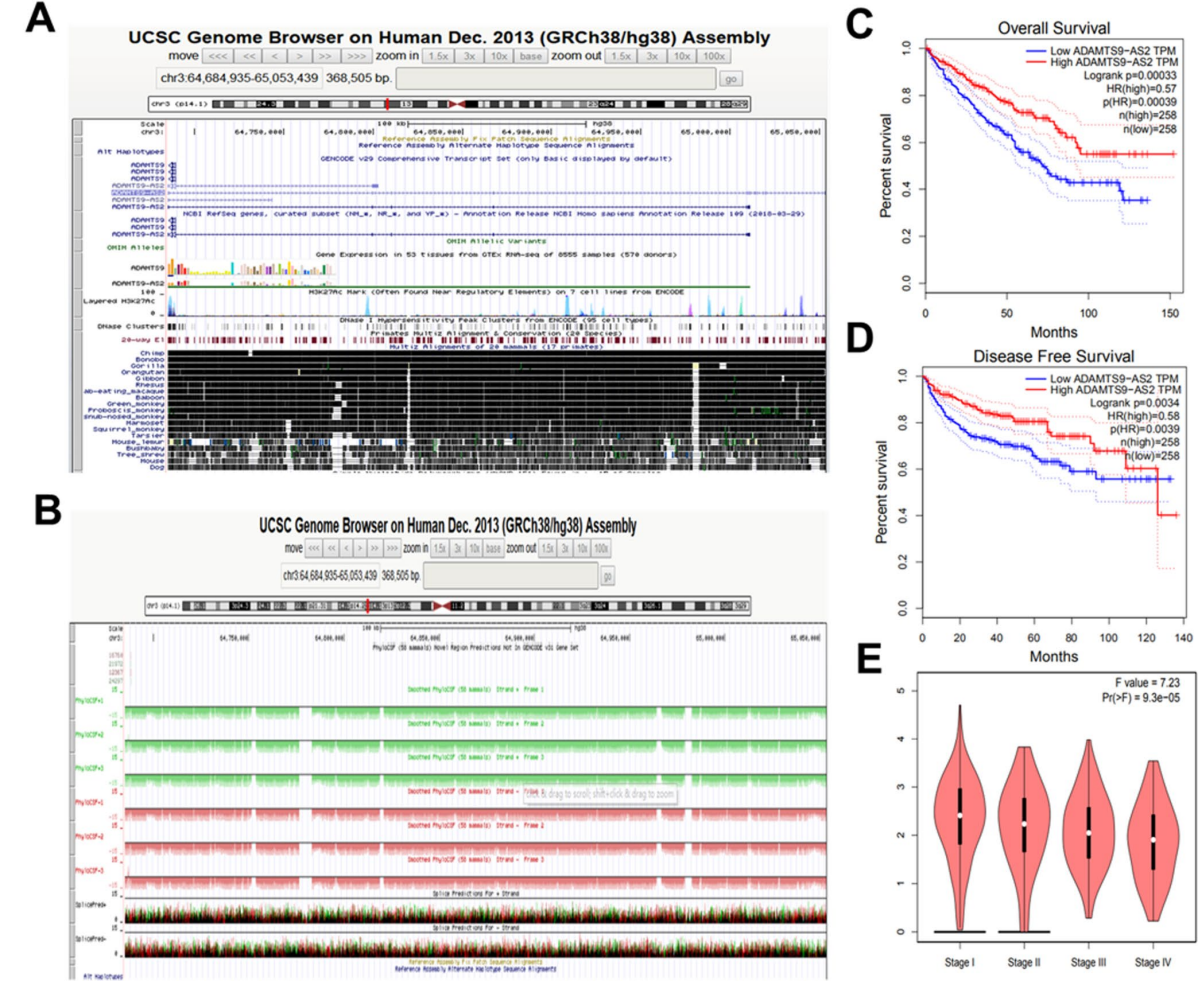
**ADAMTS9-AS2 is a lncRNA suggested by its bioinformatics analyses.** (**A**) The chromosomal location and conservation analysis of ADAMTS9-AS2 using UCSC database. (**B**) Protein-coding potential of ADAMTS9-AS2 predicted by PhyloCSF database. (**C**) Extended overall survival of patients with high expression of ADAMTS9-AS2 in KIRC. (**D**) Extended disease free survival of patients with high expression of ADAMTS9-AS2 in KIRC. (**E**) The expression level of ADAMTS9-AS2 was decreased along with KIRC stage grade increased. ADAMTS9-AS2, ADAM metallopeptidase with thrombospondin type 1 motif, 9 antisense RNA 2; lncRNA, long non-coding RNA; KIRC, kidney renal clear cell carcinoma.

According to the UCSC (hg38) database (http://genome.ucsc.edu/) analysis, ADAMTS9-AS2 is located at the positive strand of chromosome 3 (chr3: 64, 684, 935-65, 053, 439) with length of 2.258 kb. As shown in Figure 1A, ADAMTS9-AS2 is well conservative among different species, indicating its potential of regulatory functions in different species. PhyloCSF analysis suggested that all PhyloCSF values of ADAMTS9-AS2 are less than 0, indicating that ADAMTS9-AS2 does not have coding ability (Figure 1B). In addition, the Coding Potential Assessment Tool (CPAT, http://lilab.research.bcm.edu/cpat/) database further verified that ADAMTS9-AS2 has a 183 bp open reading frame but with no coding ability (Supplementary Figure 1A). As shown in Supplementary Figure 1B, no conserved domains were identified in the ADAMTS9-AS2 sequence according to Conserved Domain Database (CDD, https://www.ncbi.nlm.nih.gov/Structure/cdd/cdd.shtml). These results altogether indicate that ADAMTS9-AS2 is a lncRNA.

Next, we aimed to analyze the association of ADAMTS9-AS2 with the prognosis of kidney renal clear cell carcinoma (KIRC, also termed ccRCC) patients according to Gene Expression Profiling Interactive Analysis (GEIPA) database. Results showed that KIRC patients with high-expressed ADAMTS9-AS2 had evidently better overall and disease-free survival (Figure 1C, 1D, n=258, p=0.00039). Moreover, the expression level of ADAMTS9-AS2 was decreased along with the tumor malignancy increased (Figure 1E, *P*<0.05). These findings reveale that high-expressed ADAMTS9-AS2 is associated with better prognosis of KIRC patients, highlighting its potential to be a specific target for indicated cancer.

In order to recognize the function of ADAMTS9-AS2 in tumors, pan-cancer analysis was performed using the GEPIA database. Bodymap showed that ADAMTS9-AS2 expression was downregulated in many tumor sites of body (Supplementary Figure 2A). In addition, ADAMTS9-AS2 expression in various human tumor tissues was also significantly lower than in normal tissues (Supplementary Figure 2B). Subsequently, analyses of Kaplan-Meier (KM) plotter database showed that high-expressed ADAMTS9-AS2 was correlated with good prognosis in KIRC, lung adenocarcinoma (LUAD), liver hepatocellular carcinoma (LIHC), pheochromocytoma and paraganglioma (PCPG), sarcoma (SARC), thyroid carcinoma (THCA) and lung adenocarcinoma (LUAD) (Supplementary Figure 2C). Taken together, these findings demonstrate that ADAMTS9-AS2 may play a tumor-suppressive role in the progression and development of various human cancers, including KIRC (namely ccRCC).

### ADAMTS9-AS2 expression is clearly downregulated in ccRCC tissues and cell lines

**Figure 2:**
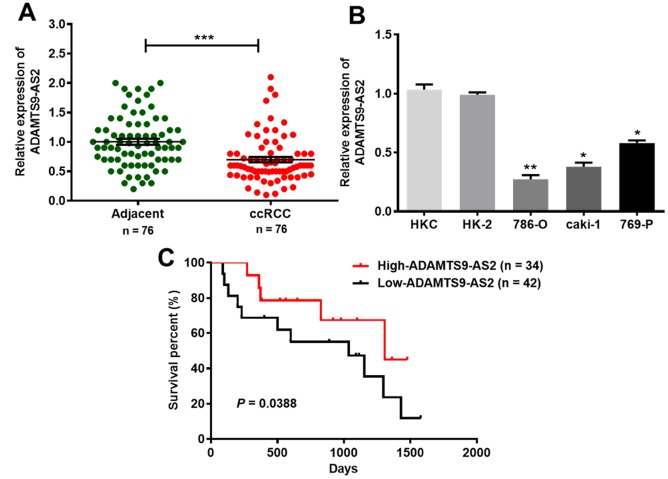
**ADAMTS9-AS2 expression is clearly downregulated in ccRCC tissues and cell lines.** (**A**) ADAMTS9-AS2 showed decreased levels of expression in 76 clinical ccRCC tissues compared to those of the normal adjacent tissues, as analyzed by qRT-PCR. (**B**) ADAMTS9-AS2 displayed significantly decreased expression levels in three ccRCC cell lines (786-O, caki-1 and 769-P) compared to those of two normal renal proximal tubular epithelial cell lines (HKC, HK-2). (**C**) Kaplan-Meier analysis of the association between ADAMTS9-AS2 expression and the survival time in 76 clinical ccRCC patients. Three independent experiments were performed and data shown are mean ± SD. Statistically significant differences are indicated as *, *P*<0.05, **, *P*<0.01, ***, *P*<0.001; Student’s *t*-test. ADAMTS9-AS2, ADAM metallopeptidase with thrombospondin type 1 motif, 9 antisense RNA 2; ccRCC, clear cell renal cell carcinoma; qRT-PCR, quantitative real-time polymerase chain reaction; SD, standard deviation.

To investigate the role of ADAMTS9-AS2 in ccRCC development, we first determined the expression levels of ADAMTS9-AS2 in 76 ccRCC tissues. ADAMTS9-AS2 showed lower levels of expression in ccRCC tissues than in normal adjacent tissues, as analyzed by quantitative real-time polymerase chain reaction (qRT-PCR) (Figure 2A, *P*<0.001). In a similar manner, we further analyzed ADAMTS9-AS2 expression in five cell lines, namely, HKC, HK-2, 786-O, caki-1 and 769-P. Compared to the two normal renal proximal tubular epithelial cell lines (HKC, HK-2), levels of ADAMTS9-AS2 expression were significantly decreased in the 786-O, caki-1 and 769-P cell lines (Figure 2B, *P*<0.05, *P*<0.01). In addition, Kaplan-Meier survival analysis was employed to investigate the association between ADAMTS9-AS2 expression and the survival of 76 clinical ccRCC patients. As revealed in Figure 2C, ccRCC patients with low-expressed ADAMTS9-AS2 had relatively short survival time, while those with high-expressed had relatively long survival time (*P*=0.0388). The correlation between ADAMTS9-AS2 expression and the clinicopathologic features of ccRCC patients is shown in Table 1. It was determined that ADAMTS-AS2 expression was correlated with tumor stage and tumor diameter (*P*<0.01) but not significantly correlated with age, gender, and Fuhrman grade. Taken together, these results demonstrate that ADAMTS9-AS2 is evidently downregulated in ccRCC, and its expression is correlated with tumor stage and tumor diameter.

**Table 1:** Correlation between ADAMTS9-AS2 expression and the clinicopathologic features of ccRCC patients.

**Factors**	**Total (n = 76)**	**Expression of ADAMTS9-AS2**		***P-*value**
		**Low**	**High**	
Age (years)				0.843
≤ 50	30	17	13	
> 50	46	25	21	
Gender				0.689
Male	41	29	12	
Female	35	13	22	
Tumor stage				0.0001**
pT1	39	12	27	
pT2/T3	37	30	7	
Fuhrman grade				0.572
Grade ½	52	27	25	
Grade ¾	24	14	10	
Tumor diameter (cm)				0.004**
≤ 7 cm	41	15	26	
> 7 cm	35	27	8	

ADAMTS9-AS2, ADAM metallopeptidase with thrombospondin type 1 motif, 9 antisense RNA 2; ccRCC, clear cell renal cell carcinoma. **P < 0.01

### ADAMTS9-AS2 binds to miR-27a-3p and inhibits its expression

**Figure 3:**
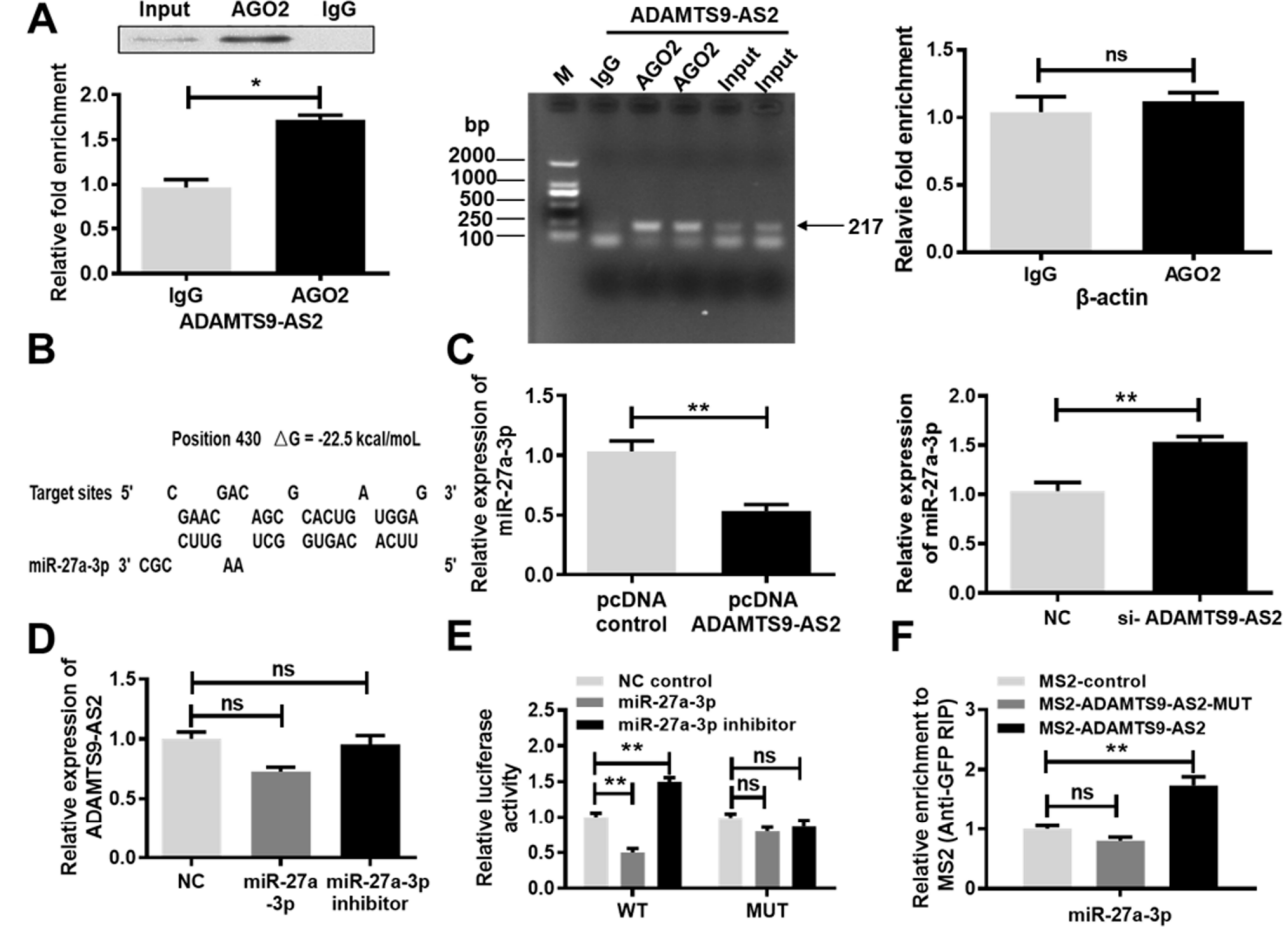
**ADAMTS9-AS2 binds to miR-27a-3p and inhibits its expression.** (**A**) ADAMTS9-AS2 was preferentially enriched in Ago2-containing beads compared to beads harboring the IgG antibody, whereas β-actin was not detectably enriched, as analyzed by RIP assay. (**B**) Schematic diagrams of the mutual interactions between miR-27a-3p and ADAMTS9-AS2. The calculated ΔG values (kcal/mol) are presented. (**C**) Overexpression of ADAMTS9-AS2 contributed to an obvious reduction of miR-27a-3p expression while knockdown led to an evident increase of miR-27a-3p expression according to qRT-PCR analysis. (**D**) Overexpression or knockdown of miR-27a-3p had no obvious effect on ADAMTS9-AS2 expression at the mRNA level, according to qRT-PCR analysis. (**E**) The luciferase reporter gene vector containing ADAMTS9-AS2 3′UTR WT or MUT with miR-27a-3p was respectively transfected into caki-1 cells. MiR-27a-3p notably suppressed the activity of the luciferase reporter harboring full length ADAMTS9-AS2 in the WT group but had no significant effect in the MUT group. (**F**) A pull-down assay was performed in caki-1 cells transfected by ADAMTS9-AS2 with the presence or absence of miR-27a-3p binding sites. Three independent experiments were performed and data shown are mean ± SD. Statistically significant differences are indicated as *, *P*<0.05, **, *P*<0.01; ns, no significance; Student’s *t*-test among two groups; ANOVA among multiple groups. ADAMTS9-AS2, ADAM metallopeptidase with thrombospondin type 1 motif, 9 antisense RNA 2; miR-27a-3p, microRNA-27a-3p; RIP, RNA immunoprecipitation; qRT-PCR, quantitative real-time polymerase chain reaction; WT, wild type; MUT, mutant type; SD, standard deviation.

LncRNAs bind to miRNAs, thereby functioning as competing endogenous RNAs, which has recently been identified as a novel posttranscriptional regulatory mechanism associated with tumorigenesis in multiple cancer types [18]. It is well known that miRNAs exert their function by binding to Ago2, a core component of the RNA-induced silencing complex (RISC). To assess whether ADAMTS9-AS2 was associated with RISC, a RNA immunoprecipitation (RIP) assay was performed using antibodies against human Ago2. Results from the RIP assay revealed that ADAMTS9-AS2 was preferentially enriched in Ago2-containing beads compared to beads harboring the control IgG antibody (Figure 3A, *P*<0.05, left and middle panels). However, negative control β-actin did not exhibit specific enrichment, suggesting no detectable association with RISC (Figure 3A, right panel). In addition, we identified miR-27a-3p as a potential target of ADAMTS9-AS2, according to LncBase Predicted v.2 and BiBiServ2 (Figure 3B). According to qRT-PCR analysis, the overexpression of ADAMTS9-AS2 led to an obvious reduction in miR-27a-3p expression (Figure 3C, *P*<0.01, left panel) while the knockdown of ADAMTS9-AS2 resulted in a significant increase in miR-27a-3p expression (Figure 3C, *P*<0.01, right panel). Before these experiments, the efficiencies of constructed pcDNA ADAMTS9-AS2 or si-ADAMTS9-AS2 were verified in 786-O and caki-1 cells. Expression of ADAMTS9-AS2 was notably upregulated in pcDNA ADAMTS9-AS2 group comparable to pcDNA group (Supplementary Figure 3A, *P*<0.01). Conversely, expression of ADAMTS9-AS2 was observably downregulated in si-ADAMTS9-AS2-1 group comparable to NC group (Supplementary Figure 3B, *P*<0.01). No significant difference, nonetheless, was observed in the expression of ADAMTS9-AS2 between si-ADAMTS9-AS2-2 and NC groups (Supplementary Figure 3B). Similar to tests of ADAMTS9-AS2 overexpression and knockdown, the efficiency of miR-27a-3p mimic or inhibitor was also confirmed in 786-O and caki-1 cells (Supplementary Figure 3C, *P*<0.05, *P*<0.01). However, neither overexpression nor knockdown of miR-27a-3p had obvious effects on ADAMTS9-AS2 expression at the mRNA level (Figure 3D). In the luciferase reporter assay, overexpression of miR-27a-3p notably suppressed the activity of luciferase reporter harboring full length ADAMTS9-AS2 in the wild type (WT) group (Figure 3E, *P* < 0.05). Conversely, site-directed mutagenesis of the binding sites successfully abolished these suppressive effects (Figure 3E). These results indicate that miR-27a-3p mediated a translational suppression-like effect rather than RNA degradation. The pull-down assay was carried out to further investigate whether ADAMTS9-AS2 and miR-27a-3p were binding partners. As shown in Figure 3F, ADAMTS9-AS2 was confirmed to bind with miR-27a-3p. Mutation of the ADAMTS9-AS2 binding sequence with miR-27a-3p inhibited miR-27a-3p precipitation. Collectively, these results pinpoint a role of ADAMTS9-AS2 as a miRNA decoy for miR-27a-3p.

### ADAMTS9-AS2 inhibits and miR-27a-3p promotes ccRCC cell proliferation

**Figure 4:**
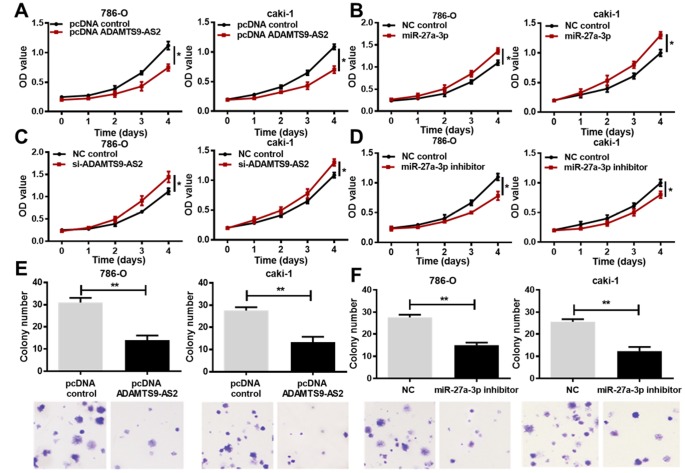
**ADAMTS9-AS2 overexpression and miR-27a-3p knockdown inhibit ccRCC cell proliferation.** (**A**) 786-O and caki-1 cells transfected with pcDNA ADAMTS9-AS2 exhibited decreased proliferation compared to those with pcDNA control, based on the MTS assay. (**B**) 786-O and caki-1 cells transfected with miR-27a-3p exhibited increased proliferation compared to cells transfected with NC, based on the MTS assay. (**C**) 786-O and caki-1 cells transfected with si-ADAMTS9-AS2 displayed preferable proliferation compared to cells transfected with NC, based on the MTS assay. (**D**) 786-O and caki-1 cells transfected with miR-27a-3p inhibitor exhibited decreased proliferation compared to cells transfected with NC, based on the MTS assay. (**E**) Colony formation and crystal violet staining assays were performed in 786-O and caki-1 cells transfected with pcDNA control or pcDNA ADAMTS9-AS2, or (**F**) in 786-O and caki-1 cells transfected with NC or miR-27a-3p inhibitor. ADAMTS9-AS2 overexpression and miR-27a-3p knockdown significantly repressed colony formation of ccRCC cells. Scale bar = 100 μm. Three independent experiments were performed and data shown are mean ± SD. Statistically significant differences are indicated as *, *P*<0.05, **, *P*<0.01; Student’s *t*-test. ADAMTS9-AS2, ADAM metallopeptidase with thrombospondin type 1 motif, 9 antisense RNA 2; miR-27a-3p, microRNA-27a-3p; MTS, (3-(4,5-dimethylthiazol-2-yl)-5-(3-carboxymethoxyphenyl)-2-(4-sulfop-henyl)-2H-tetrazolium); si, small interfering; NC, negative control; ccRCC, clear cell renal cell carcinoma; SD, standard deviation.

To investigate the impact of ADAMTS9-AS2 or miR-27a-3p on ccRCC, cell proliferation was subsequently analyzed by 3-(4, 5-dimethylthiazol-2- yl)-5-(3-carboxymethoxyphenyl)-2-(4-sulfop-henyl)-2H-tetrazolium (MTS) assay in both 786-O and caki-1 cells after ADAMTS9-AS2/miR-27a-3p overexpression or knockdown. The pcDNA-ADAMTS9-AS2 was constructed and transfected into cells for ADAMTS9-AS2 overexpression, while small interfering (si)-ADAMTS9-AS2 was transfected into cells for ADAMTS9-AS2 knockdown. The miR-27a-3p mimic or inhibitor was respectively transfected into cells for miR-27a-3p overexpression or knockdown. Cell proliferation in both cell lines (789-O, caki-1) was significantly inhibited by ADAMTS9-AS2 overexpression (Figure 4A, *P*<0.05). Contrary to ADAMTS9-AS2 overexpression, miR-27a-3p overexpression in 786-O and caki-1 cells facilitated cell proliferation (Figure 4B, *P*<0.05). In the knockdown experiments, ADAMTS9-AS2 knockdown led to the increased proliferation of ccRCC cells, which was the opposite result of that obtained with the miR-27a-3p knockdown (Figure 4C, 4D, *P*<0.05). Consistently, we observed that the colony formation of ccRCC cells was significantly inhibited in 786-O and caki-1 cells with ADAMTS9-AS2 overexpression (Figure 4E, *P*<0.01) or miR-27a-3p knockdown (Figure 4F, *P*<0.01), according to the crystal violet staining. These data collectively indicate that ADAMTS9-AS2 has an inhibitory role and miR-27a-3p has a stimulative role in ccRCC cell proliferation.

### ADAMTS9-AS2 reduces ccRCC cell chemoresistance

**Figure 5:**
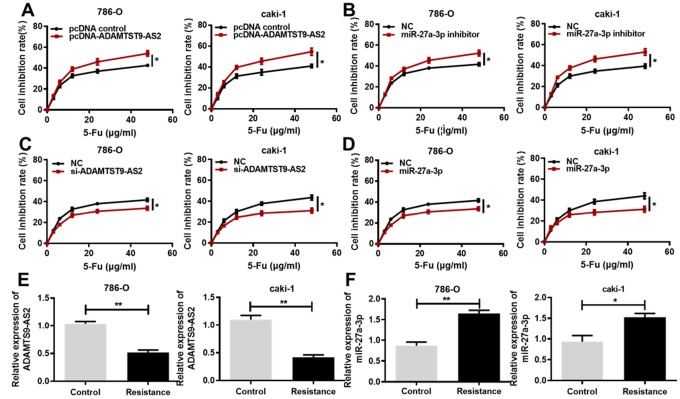
**ADAMTS9-AS2 overexpression and miR-27a-3p knockdown decrease ccRCC cell chemoresistance to 5-Fu.** (**A**) MTS assays were performed in 786-O and caki-1 cells transfected with pcDNA control or pcDNA ADAMTS9-AS2 and treated with the indicated concentrations of 5-Fu. (**B**) MTS assays were performed in 786-O and caki-1 cells transfected with NC or miR-27a-3p inhibitor and treated with the indicated concentrations of 5-Fu. (**C**) MTS assays were performed in 786-O and caki-1 cells transfected with NC or si-ADAMTS9-AS2 and treated with the indicated concentrations of 5-Fu. (**D**) MTS assays performed in 786-O and caki-1 cells transfected with NC or miR-27a-3p and treated with the indicated concentrations of 5-Fu. (**E**) Expression levels of ADAMTS9-AS2 and (**F**) miR-27a-3p were determined by qRT-PCR in 5-Fu-resistant 786-O and caki-1 cells. Three independent experiments were performed and data shown are mean ± SD. Statistically significant differences are indicated as *, *P*<0.05, **, *P*<0.01; Student’s *t*-test. ADAMTS9-AS2, ADAM metallopeptidase with thrombospondin type 1 motif, 9 antisense RNA 2; miR-27a-3p, microRNA-27a-3p; ccRCC, clear cell renal cell carcinoma; MTS, (3-(4,5-dimethylthiazol-2-yl)-5-(3-carboxymethoxyphenyl)-2-(4-sulfop-henyl)-2H-tetrazolium); si, small interfering; NC, negative control; qRT-PCR, quantitative real-time polymerase chain reaction; SD, standard deviation.

Based on previous research of noncoding RNAs involved in the chemoresistance of cancer cells [19], we hypothesized that ADAMTS9-AS2 and miR-27a-3p regulate the chemoresistance of ccRCC cells. To verify this hypothesis, we treated ccRCC cells that were transfected with ADAMTS9-AS2 overexpression or miR-27a-3p knockdown with a series of increasing concentrations of 5-Fu. As expected, 5-Fu led to the inhibition of cell proliferation in a dose-dependent manner. We found that the sensitivity of ccRCC cells to 5-Fu treatment was increased by either ADAMTS9-AS2 overexpression (Figure 5A, *P*<0.05) or miR-27a-3p knockdown (Figure 5B, *P*<0.05), and thus cell proliferation was inhibited. Conversely, ADAMTS9-AS2 knockdown (Figure 5C, *P*<0.05) and miR-27a-3p overexpression (Figure 5D, *P*<0.05) resulted in the insensitivity of ccRCC cells to increased 5-Fu and, moreover, partially alleviated the growth inhibition of ccRCC cells. Importantly, the expression levels of ADAMTS9-AS2 were significantly decreased in the resistance group (namely, chemoresistant ccRCC cells) compared to the control group (Figure 5E, *P*<0.01), whereas the opposite results were obtained with the expression levels of miR-27a-3p (Figure 5F, *P*<0.05). These results were further confirmed with the treatment of another chemotherapy drug, Cisplatin. Consistently, ADAMTS9-AS2 overexpression (Supplementary Figure 4A, *P*<0.05) and miR-27a-3p knockdown (Supplementary Figure 4B, *P*<0.05) led to the increased sensitivity of ccRCC cells to Cisplatin treatment, and both ADAMTS9-AS2 knockdown (Supplementary Figure 4C, *P*<0.05) and miR-27a-3p overexpression (Supplementary Figure 4D, *P*<0.05) contributed to the decreased sensitivity of ccRCC cells to Cisplatin. Importantly, we also found that in chemoresistant ccRCC cells, the expression levels of ADAMTS9-AS2 were significantly decreased (Supplementary Figure 4E, *P*<0.01), whereas those of miR-27a-3p were increased (Supplementary Figure 4F, *P*<0.05, *P*<0.01). Taken together, these data indicate that ADAMTS9-AS2 attenuates the resistance of ccRCC cells to chemotherapy drugs.

### MiR-27a-3p targets FOXO1 and inhibits its expression

**Figure 6:**
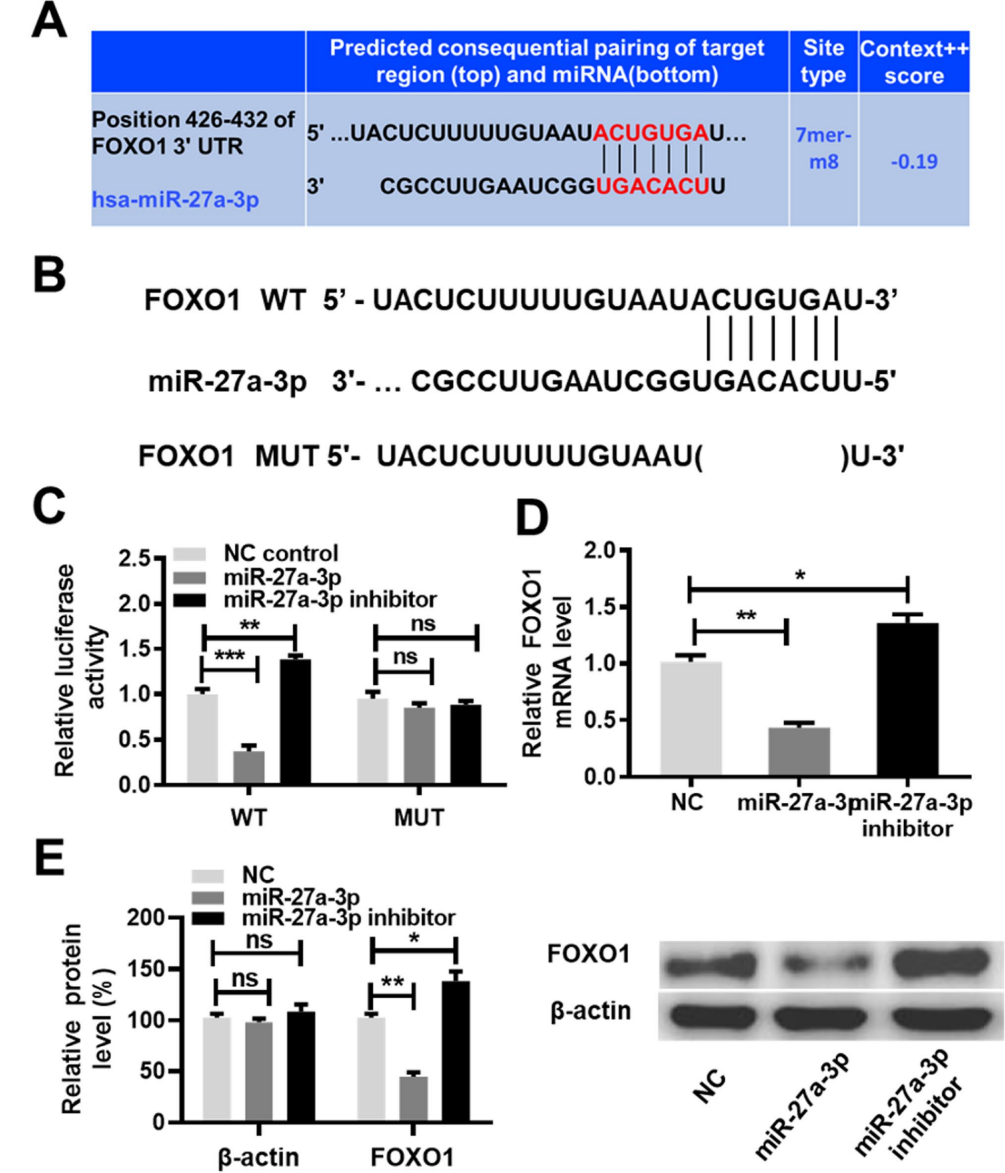
**MiR-27a-3p targets FOXO1 and inhibits its expression.** (**A**) Predicted consequential pairing of the target region (FOXO1 3′UTR) and miR-27a-3p from TargetScan (http://www.targetscan.org/vert_72/). (**B**) Schematic illustration of the predicted miR-27a-3p binding sites with the 3′UTR of FOXO1. (**C**) A luciferase activity assay was performed in caki-1 cells co-transfected with miR-27a-3p and the luciferase reporter plasmids driven by either WT or MUT 3′UTR of FOXO1. (**D**) The expression of FOXO1 at the mRNA level was determined by qRT-PCR analysis in 786-O and caki-1 cells transfected with NC, miR-27a-3p, or miR-27a-3p inhibitor. (**E**) The expression of FOXO1 at the protein level was determined by western blot analysis in 786-O and caki-1 cells transfected with NC, miR-27a-3p, or miR-27a-3p inhibitor. Three independent experiments were performed and data shown are mean ± SD. Statistically significant differences are indicated as *, *P*<0.05, **, *P*<0.01; ns, no significance; Student’s *t*-test among two groups; ANOVA among multiple groups. miR-27a-3p, microRNA-27a-3p; FOXO1, Forkhead Box Protein O1; UTR, untranslated region; WT, wild type; MUT, mutant type; qRT-PCR, quantitative real-time polymerase chain reaction; NC, negative control; SD, standard deviation.

Two publicly available algorithms (DIANA TOOLS and TargetScan) were used to identify the potential targets of miR-27a-3p. As presented by TargetScan, miR-27a-3p was predicted to target the FOXO1 gene (Figure 6A). Previous studies have reported that miR-27a-3p promotes cell proliferation by targeting FOXO1 [20, 21]. The function of FOXO1 in the chemoresistance in cancer cells has been evaluated *in vitro* [22, 23]. Hence, we sought to confirm this prediction in the context of ccRCC cells. Based on the predicted binding sites of miR-27a-3p, FOXO1 3′ untranslated region (3′UTR) WT and mutant type (MUT) luciferase reporter plasmids were generated (Figure 6B). A luciferase reporter assay was performed by co-transfecting the luciferase reporter plasmids with miR-27a-3p, miR-27a-3p inhibitor or NC control, respectively. As shown in Figure 6C, the overexpression of miR-27a-3p decreased the luciferase activity driven by the WT 3′UTR of FOXO1 (*P* < 0.01), but no obvious alteration in the luciferase activity driven by the MUT 3′UTR of FOXO1 was observed. Furthermore, FOXO1 expression at both the mRNA and protein levels were reduced by miR-27a-3p overexpression and increased by miR-27a-3p knockdown in ccRCC cells, as determined by qRT-PCR and western blot assays (Figure 6D, 6E, *P*<0.05, *P*<0.01). These results indicate that miR-27a-3p targets FOXO1 and inhibits its expression.

### ADAMTS9-AS2 impedes ccRCC cell proliferation and decreases chemoresistance by acting as a miR-27a-3p sponge

**Figure 7:**
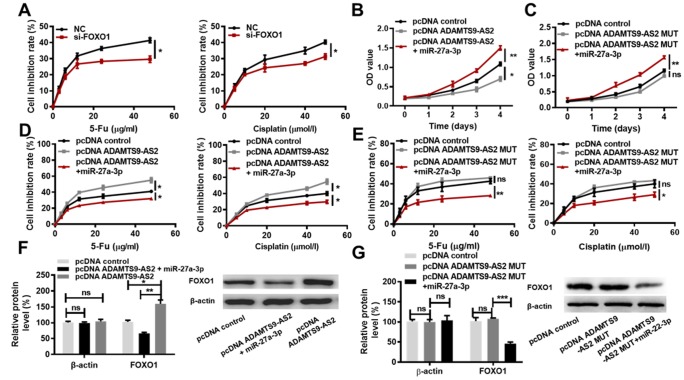
**ADAMTS9-AS2 impedes ccRCC cell proliferation and chemoresistance by acting as a miR-27a-3p sponge.** (**A**) MTS assays were performed in caki-1 cells transfected with NC or si-FOXO1 and treated with the indicated concentrations of 5-Fu or Cisplatin. (**B**) MTS assays were performed in caki-1 cells transfected with pcDNA control, pcDNA ADAMTS9-AS2, or pcDNA ADAMTS9-AS2 simultaneous with miR-27a-3p, respectively. (**C**) MTS assays were performed in caki-1 cells transfected with pcDNA control, pcDNA ADAMTS9-AS2 MUT or pcDNA ADAMTS9-AS2 MUT simultaneous with miR-27a-3p, respectively. (**D**) MTS assays were performed in caki-1 cells transfected with pcDNA control, pcDNA ADAMTS9-AS2, or pcDNA ADAMTS9-AS2 simultaneous with miR-27a-3p and treated with the indicated concentrations of 5-Fu or Cisplatin. (**E**) MTS assays were performed in caki-1 cells transfected with pcDNA control, pcDNA ADAMTS9-AS2 MUT, or pcDNA ADAMTS9-AS2 MUT simultaneous with miR-27a-3p and treated with the indicated concentrations of 5-Fu or Cisplatin. (**F**) The expression of FOXO1 at the protein level was determined in caki-1 cells transfected with pcDNA control, pcDNA ADAMTS9-AS2, or pcDNA ADAMTS9-AS2 simultaneous with miR-27a-3p by western blot analysis. (**G**) The expression of FOXO1 at protein level was determined in caki-1 cells transfected with pcDNA control, pcDNA ADAMTS9-AS2 MUT or pcDNA ADAMTS9-AS2 MUT simultaneous with miR-27a-3p by western blot analysis. Three independent experiments were performed and data shown are mean ± SD. Statistically significant differences are indicated as *, *P*<0.05, **, *P*<0.01, ***, *P*<0.001; ns, no significance; Student’s *t*-test among two groups; ANOVA among multiple groups. ADAMTS9-AS2, ADAM metallopeptidase with thrombospondin type 1 motif, 9 antisense RNA 2; ccRCC, clear cell renal cell carcinoma; miR-27a-3p, microRNA-27a-3p; MTS, 3-(4,5-dimethylthiazol-2-yl)-5-(3-carboxy-methoxyphenyl)-2-(4-sulfop-henyl)-2H-tetrazoli-um); NC, negative control; si, small interfering; FOXO1, Forkhead Box Protein O1; MUT, mutant type; SD, standard deviation.

As mentioned above, ADAMTS9-AS2 overexpression and miR-27a-3p knockdown contribute to the inhibition of ccRCC cell proliferation and chemoresistance. Since ADAMTS9-AS2 inhibited miR-27a-3p activity, and miR-27a-3p inhibited FOXO1 expression, we hypothesized that the inhibition of ccRCC cell proliferation and chemoresistance induced by ADAMTS9-AS2 overexpression may be attributed to the decreased expression of miR-27a-3p and consequent increased expression of FOXO1. To test this hypothesis, we knocked down FOXO1 and performed MTS cell proliferation assays in the presence of 5-Fu or Cisplatin. Two siRNAs against FOXO1 (si-FOXO1-1, si-FOXO1-2) were constructed, and their efficiencies were determined in both 786-O and caki-1 cells. Obviously, si-FOXO1-1 showed a higher inhibitory effect on FOXO1 expression than si-FOXO1-2 in 786-O and caki-1 cells when compared to NC group (Supplementary Figure 3D, *P*<0.05, *P*<0.01). si-FOXO1-1 was therefore used for the following tests. As shown in Figure 7A, cells in the presence of 5-Fu or Cisplatin exhibited lower chemoresistance in the si-FOXO1 group compared to the NC group (*P* < 0.05). While ADAMTS9-AS2 overexpression led to significant inhibition of ccRCC cell proliferation, the simultaneous overexpression of miR-27a-3p completely reversed this inhibition (Figure 7B, *P* < 0.05). ADAMTS9-AS2 overexpression MUT had no significant inhibitory effect on ccRCC cell proliferation, whereas the simultaneous miR-27a-3p overexpression promoted cell proliferation (Figure 7C, *P*<0.01). This finding indicates that the decreased levels of miR-27a-3p expression were essential for the inhibition of cell proliferation induced by ADAMTS9-AS2 overexpression.

Regarding to the regulation of ccRCC cell chemoresistance by ADAMTS9-AS2, we found that the increased sensitivity of ccRCC cells to both 5-Fu and Cisplatin treatment induced by ADAMTS9-AS2 overexpression was completely abolished by the simultaneous overexpression of miR-27a-3p (Figure 7D, *P*<0.05). ADAMTS9-AS2 overexpression MUT had no significant facilitating effect on sensitivity of ccRCC cells to both 5-Fu and Cisplatin treatment, whereas the simultaneous miR-27a-3p overexpression decreased the sensitivity of ccRCC cells to both 5-Fu and Cisplatin treatment (Figure 7E, *P*<0.01). These results indicated that the decreased levels of miR-27a-3p expression were required for the increased sensitivity of ccRCC cells to chemotherapy drugs induced by ADAMTS9-AS2 overexpression. In addition, we sought to determine whether the ADAMTS9-AS2-mediated regulation of FOXO1 expression in ccRCC cells was dependent on miR-27a-3p expression levels. As expected, the overexpression of ADAMTS9-AS2 led to increased protein levels of FOXO1 (Figure 7F, *P*<0.05). However, the simultaneous overexpression miR-27a-3p reversed the increase in FOXO1 expression at protein level (Figure 7F, *P*<0.01). ADAMTS9-AS2 overexpression MUT had no marked effect on protein levels of FOXO1, while the simultaneous overexpression miR-27a-3p markedly decreased FOXO1 expression at protein level (Figure 7G, *P*<0.01). These findings indicate that ADAMTS9-AS2 promotes FOXO1 expression in ccRCC cells via constraining miR-27a-3p expression. Collectively, these data strongly support the hypothesis that ADAMTS9-AS2 impedes ccRCC cell proliferation and decreases chemoresistance via acting as a miR-27a-3p sponge.

## DISCUSSION

Despite significant progress in the treatment of ccRCC, patients have varying degrees of sensitivity to the available chemotherapeutics, due to tumor heterogeneity, which predicts poor prognosis and outcome in patients. In this context, better therapeutic approaches for ccRCC remain a public health problem. LncRNAs that act as oncogenes or tumor-suppressor genes play a crucial, even decisive, role in tumorigenesis and cancer development. For example, lncRNA GAS5 induces cell apoptosis in breast cancer has been reported [6]. Yang X. *et. al*. demonstrated that lncRNA HNF1A-AS1 is strongly associated with the proliferation and migration of esophageal adenocarcinoma cells [14]. In RCC, many lncRNAs, including TUG1 [11], HEIRCC [12] and SRLR [24], have been reported to be aberrantly expressed and correlated with proliferation and apoptosis, revealing the critical roles of lncRNAs in RCC etiology. According to our bioinformatics analyses, ADAMTS9-AS2 is located at the positive strand of chromosome 3 (chr3: 64, 684, 935-65, 053, 439) with length of 2.258 kb and belongs to lncRNA. It has also been demonstrated that ADAMTS9-AS2 is downregulated in colorectal cancer, and moreover, its low expression was found to be associated with poor prognosis in patients [25]. However, little is known about the functional role and potential mechanisms of ADAMTS9-AS2 in ccRCC.

In the current study, bioinformatics analyses indicated that high-expressed ADAMTS9-AS2 is associated with better prognosis of KIRC (namely ccRCC) patients and downregulated in various tumor tissues including ccRCC. Consistent with these results, we identified significant downregulation of ADAMTS9-AS2 in ccRCC tissues and cell lines. Moreover, low expression of ADAMTS9-AS2 was associated with poor patient outcomes in primary ccRCC. This phenomenon inspired us to speculate on the key role of ADAMTS9-AS2 in the progression of ccRCC.

As previously reported, ADAMTS9-AS2 was shown to have an inhibitory role in NSCLC cell proliferation and migration, unearthing the potential tumor-suppressor role of this lncRNA in the development and progression of NSCLC [16]. Based on this result, we used gain-of-function and loss-of-function assays to determine that ADAMTS9-AS2 suppressed cell proliferation and reduced chemoresistance in ccRCC, implying that ADAMTS9-AS2 may act as a tumor suppressor in ccRCC.

During the initiation and development of various cancers, miRNA expression undergoes evident alteration [23, 26]. Discussions regarding the possibility of therapeutically targeting miRNAs as part of cancer treatment have dominated research in recent years. miR-27a-3p has been shown to be upregulated in many cancers, including ccRCC [26–28], and this miRNA may be an independent predictive factor for recurrence in ccRCC [26]. Furthermore, miR-27a-3p promotes cell proliferation by targeting FOXO1 [20, 21]. The results obtained from the luciferase reporter assay and pull-down assay revealed that ADAMTS9-AS2 bound to miR-27a-3p and inhibited its expression. In other words, these findings indicate a role for ADAMTS9-AS2 as a miRNA decoy for miR-27a-3p. In addition, results also revealed that miR-27a-3p targeted FOXO1 and inhibited its expression. We hypothesized that the inhibition of ccRCC cell proliferation and reduction of chemoresistance induced by ADAMTS9-AS2 overexpression may be attributed to the decreased expression of miR-27a-3p and consequent increased expression of FOXO1. We confirmed this hypothesis with a series of assays. Analytically, the essential role of miR-27a-3p in the regulation of cell proliferation, sensitivity of ccRCC cells to chemotherapy drugs, and FOXO1 expression induced by ADAMTS9-AS2 overexpression was associated with the downregulation of miR-27a-3p. These findings all indicate that ADAMTS9-AS2 impeded ccRCC cell proliferation and reduced chemoresistance by acting as a miR-27a-3p sponge.

Collectively, this study innovatively unearths that ADAMTS9-AS2 may act as an independent prognostic predictor for ccRCC diagnosis. Additionally, this study also revealed the inhibitory role of ADAMTS9-AS2 in ccRCC via the miR-27a-3p-mediated regulation of FOXO1. In fact, the regulatory role of lncRNAs in FOXO1 expression has been investigated in various studies [20, 29]. Moreover, Park J *et. al*. verified that FOXO1 is involved in drug resistance via the phosphoinositide 3-kinase/Akt pathway in gastric cancer [23]. In this context, we predict that ADAMTS9-AS2 may exert its influence in ccRCC through the phosphoinositide 3-kinase/Akt pathway, which will be addressed in future studies. These results expand our knowledge on the specific mechanism of action of ADAMTS9-AS2 in ccRCC.

## MATERIALS AND METHODS

### Protein-coding potential predictions

The genomic location of ADAMTS9-AS2 and its conservation were analyzed by the UCSC database (http://genome.ucsc.edu/). The protein coding ability of ADAMTS9-AS2 was first analyzed by UCSC combined with PhyloCSF (https://github.com/mlin/PhyloCSF/wiki), and further verified by CPAT database (http://lilab.research.bcm.edu/cpat/). The CDD (https://www.ncbi.nlm.nih.gov/Structure/cdd/cdd.shtml) was employed to predict structural conservation and stable RNA secondary structure in the thermodynamics of ADAMTS9-AS2.

### Expression and Survival analysis

The GEPIA database (http://gepia.cancer-pku.cn/) was used to analyze the expression levels of ADAMTS9-AS2 in different types of cancers. Moreover, the association of expression profile of ADAMTS9-AS2-high vs ADAMTS9-AS2-low with the overall survival or with the disease-free survival or with varied stages of ccRCC patients was also evaluated. The KM Plotter (http://kmplot.com/analysis/) database was used to further analyze the association of ADAMTS9-AS2 expression with the overall survival in different types of cancer patients.

### Tissue specimens

Samples from ccRCC tumors and paired normal adjacent tissues were collected from patients (n = 76) with ccRCC who were undergoing an initial operation at the First Affiliated Hospital of Harbin Medical University (Heilongjiang, China). The case-matched normal tissues were obtained at least 5 cm from the edge of the lesion. All samples were immediately snap-frozen in liquid nitrogen after the resection and then maintained in storage. All of the ccRCC cases were clinically and pathologically confirmed and staged based on the 2009 Union for International Cancer Control TNM (version 7) classification of malignant tumors. The clinical characteristics of 76 ccRCC patients are shown in Table 1. Written informed consent was obtained from all patients. The utilization of these samples was approved by the Ethical Committee of First Affiliated Hospital of Harbin Medical University (Heilongjiang, China).

### Cell culture and transfection

The cell culture conditions and relevant reagents were described in our previous paper [30]. Three ccRCC cell lines (786-O, caki-1 and 769-P) and normal renal proximal tubular epithelial cell lines (HKC, HK-2) were cultured in Dulbecco’s Modified Eagle’s Medium (HyClone, Logan, USA) containing 10% fetal bovine serum (Thermo Fisher Scientific, Inc., Waltham, MA, USA) in a sterile incubator at 37°C with 5% CO_2_ (v/v).

To generate the ADAMTS9-AS2 overexpression cell lines, pcDNA-ADAMTS9-AS2 was constructed by Sangon Biotech (Shanghai, China). To generate the ADAMTS9-AS2 knockdown models, two siRNAs (RiboBio, Guangzhou, China) against ADAMTS9-AS2 (si-ADAMTS9-AS2-1, si-ADAMTS9-AS2-2) were respectively transfected into 786-O and caki-1 cells to generate 786-O/si-ADAMTS9-AS2-1, 786-O/si-ADAMTS9-AS2-2, caki-1/si-ADAMTS9- AS2-1 or caki-1/si-ADAMTS9-AS2-2. To generate the miR-27a-3p overexpression cell lines, the miR-27a-3p mimic (RiboBio, Guangzhou, China) was transfected into 786-O and caki-1 cells to generate 786-O/ miR-27a-3p and caki-1/ miR-27a-3p. To generate the miR-27a-3p knockdown models, a miR-27a-3p inhibitor (RiboBio, Guangzhou, China) was transfected into 786-O and caki-1 cells to generate a 786-O/miR-27a-3p inhibitor and a caki-1/miR-27a-3p inhibitor. To generate the FOXO1 knockdown models, two siRNAs (RiboBio, Guangzhou, China) against FOXO1 (si-FOXO1-1, si-FOXO1-2) were transfected into 786-O and caki-1 cells to generate 786-O/si-FOXO1-1, 786-O/si-FOXO1-2, caki-1/si-FOXO1-1 or caki-1/si-FOXO1-2. The sequences used in this work are presented in Supplementary Table 1.

### RNA isolation and qRT-PCR analysis

The extraction of total RNA and the qRT-PCR analysis were conducted as previously described [31]. β-actin was used as the internal control for the normalization of the lncRNA data. U6 was used as the internal control for the normalization of the miRNA data. Relative gene expression was calculated using the 2^-ΔΔCt^ method. The sequences of ADAMTS9-AS2 employed in this study were in accordance with the previous study [16] and are listed in Supplementary Table 1.

### Western blot analysis

Western blot analysis was performed using the standard procedure [32]. The collected lysates were separated by 10% SDS–PAGE gel (Sangon Biotech, Shanghai, China) and transferred onto polyvinylidene fluoride membranes (Millipore, Billerica, MA). The membranes were blocked in Tris-Buffered Saline plus 0.1% Tween-20 (TBST) containing 5% nonfat dried milk for 1 h at room temperature, and then they were incubated overnight with primary antibodies (FOX1, AGO2, 1:1000, ABclonal) at 4°C. Next, after being washed 4 times with TBST, the membranes were incubated with the corresponding horseradish peroxidase-conjugated secondary antibody (goat anti-mouse, 1:10000, ABclonal) for 60 min at room temperature. Signals were detected with a chemiluminescence kit (Pierce, Thermo Fisher Scientific, Inc., USA) using medical X-ray films, and the signal intensity was quantified using Photoshop (Adobe software). β-actin served as the loading control.

### Establishment of 5-Fu-resistant cells

5-Fu-resistant ccRCC cells were generated by continuous exposure to increasing concentrations of 5-Fu (0, 3, 6, 12, 24, 48 μg/ml) with repeated subculturing until complete resistance was established. Cells were initially cultured in growing medium with 5-Fu at an initial concentration of 3 μg/ml for two months, followed by a doubling of the concentration every two months thereafter.

### Cell proliferation assay

Cell proliferation was assessed by MTS assay (Promega, Madison, WI, USA) according to the manufacturer’s protocol. A total of 1×10^3^ cells were seeded into 96-well plates in 100 μL of 10% FBS/medium and incubated at 37°C with 5% CO_2_. After incubation for 24, 48, 72, 96, and 120 h, 20 ml of CellTiter 96 Aqueous One Solution (Promega, Madison, WI, USA) was added to each well, followed by incubation for 1 h at 37°C with 5% CO_2_. Absorbance at 490 nm was measured using a microplate reader (Thermo Fisher Scientific, Inc., Waltham, MA, USA). Each experiment was performed in triplicate.

### Colony formation assay

786-O and caki-1 cells (5 × 10^2^ cells per well) were seeded in a 6-well plate and cultured for 10 days after treatment. Colonies were then fixed with 10% formaldehyde for 10 min, followed by staining for 5 min with 0.5% crystal violet. The number of colonies was determined using ImageJ. Images were visualized with an Olympus microscope (Tokyo, Japan).

### Dual-luciferase reporter assay

A fragment of WT ADAMTS9-AS2 with potential miR-27a-3p binding sites or MUT ADAMTS9-AS2 with nonfunctional binding sites was generated and inserted into the luciferase reporter vector psi-CHECK-2 (Promega, Madison, WI, USA). The full-length WT 3′UTR, containing the predicted miR-27a-3p targeting site, and the MUT 3′UTR of FOXO1 were amplified and cloned into the psi-CHECK-2 vector. Caki-1 cells were seeded on a 24-well plate and grown until they were 80% confluent. The cells were then cotransfected with the luciferase plasmid and miR-27a-3p, miR-27a-3p-inhibitor or the NC control using Lipofectamine 2000 (Invitrogen, MA, USA). The related transfection concentration of miR-27a-3p was 50 nM, according to the manufacturer’s protocol. At 48 h posttransfection, the relative luciferase activity was measured by normalizing the firefly luminescence to the Renilla luminescence using the Dual-Luciferase Reporter Assay System (Promega, Madison, WI, USA).

### RIP assay

Caki-1 cells were rinsed with cold PBS and fixed with 1% formaldehyde for 10 min. After centrifugation, the cell pellets were collected and resuspended in NP-40 lysis buffer supplemented with 1 mM PMSF, 1 mM DTT, 1% protease inhibitor cocktail (Sigma-Aldrich, USA) plus 200 U/ml RNase Inhibitor (Life Technologies, USA). The cell lysate was stored at −80°C before use. The supernatant from the cell lysate was collected by high-speed centrifugation. To generate antibody-coated beads, a Protein G Sepharose 4 Fast Flow bead slurry (GE Healthcare, USA) was rinsed with NT2 buffer (50 mM Tris-HCl, 150 mM NaCl, 1 mM MgCl_2_, 0.5% NP-40) and then incubated with an antibody against Ago2 (Abcam, UK). The mouse immunoglobulin G (IgG, Sigma-Aldrich, USA) was used as a negative control. For the RIP, the supernatant was incubated overnight with the antibody-coated Sepharose beads. Then, the beads were rinsed with cold NT2 buffer, followed by incubation with 10 mg/ml proteinase K (Sigma-Aldrich, USA). The RNA bound to the Ago2 antibody was extracted using TRIzol reagent (Invitrogen, MA, USA).

A pull-down assay was performed as previously described [33]. Caki-1 cells were co-transfected with pcDNA-MS2, pcDNA-MS2-ADAMTS9-AS2, pcDNA-MS2-AD AMTS9-AS2-MUT (miR-27a-3p) and pMS2-GFP (Addgene), respectively. After 48 h incubation, cells were used to perform RIP experiments using a GFP antibody (Roche) and the Magna RIP™ RNA-Binding Protein. The RNA bound to GFP antibody was extracted with TRIzol reagent (Invitrogen, MA, USA). MiR-27a-3p expression was examined by qRT-PCR.

### Statistical analyses

All experiments were conducted three times independently. All data are presented as the mean ± SD. Comparisons between categorical variables were assessed using a chi-squared test or Fisher’s exact test. A Student’s *t*-test or ANOVA was used to analyze the statistical significance among groups using GraphPad Prism 8.0 (La Jolla, CA, USA) according to the instruction of STATISTICS WITH PRISM 8 (https://www.graphpad.com/guides/prism/8/statistics/index.htm). The median method was used to define the high and low expression groups. The Kaplan-Meier estimator and the log-rank test were used to conduct the survival analysis. *P* < 0.05 was considered to be statistically significant.

## CONCLUSIONS

In summary, an inhibitory role of ADAMTS9-AS2 in the proliferation and its lessened role in the chemoresistance was revealed in ccRCC. In addition, the ADAMTS9-AS2 - miR-27a-3p - FOXO1 axis was identified as an aspect of the underlying mechanism. Future research should consider the functional role of FOXO1 in chemoresistance and the concrete mechanism of ADAMTS9-AS2 (such as the phosphoinositide 3-kinase/Akt pathway) in the pathogenesis of ccRCC.

## Supplementary Material

Supplementary Figures

Supplementary Table 1
