# Supplementary material for: LncRNA ADAMTS9-AS2 inhibits cell proliferation and decreases chemoresistance in clear cell renal cell carcinoma via the miR-27a-3p/FOXO1 axis
Source: Aging (Albany NY). 2019 Aug 10;11(15):5705–25. doi: 10.18632/aging.102154 (PMC6710069; doi:10.18632/aging.102154)
Supplement: Supplementary Figures [file aging-11-102154-s002.pdf]

SUPPLEMENTARY FIGURES

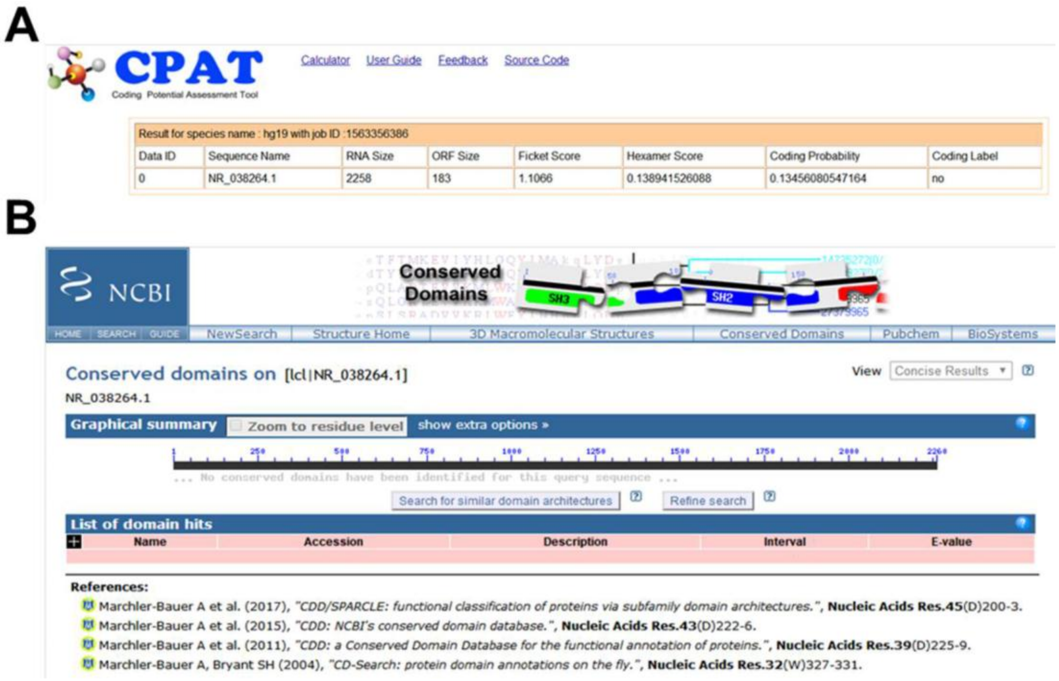

Supplementary Figure 1. Protein-coding potential and putative conserved domains of ADAMTS9-AS2 was evaluated by bioinformatics analyses. (**A**) Protein-coding potential of ADAMTS9-AS2 predicted by CPAT database. (**B**) Putative conserved domains of ADAMTS9-AS2 was forecasted by CDD analysis. ADAMTS9-AS2, ADAM metallopeptidase with thrombospondin type 1 motif, 9 antisense RNA 2; CPAT, coding potential assessment tool; CDD: conserved domains database.

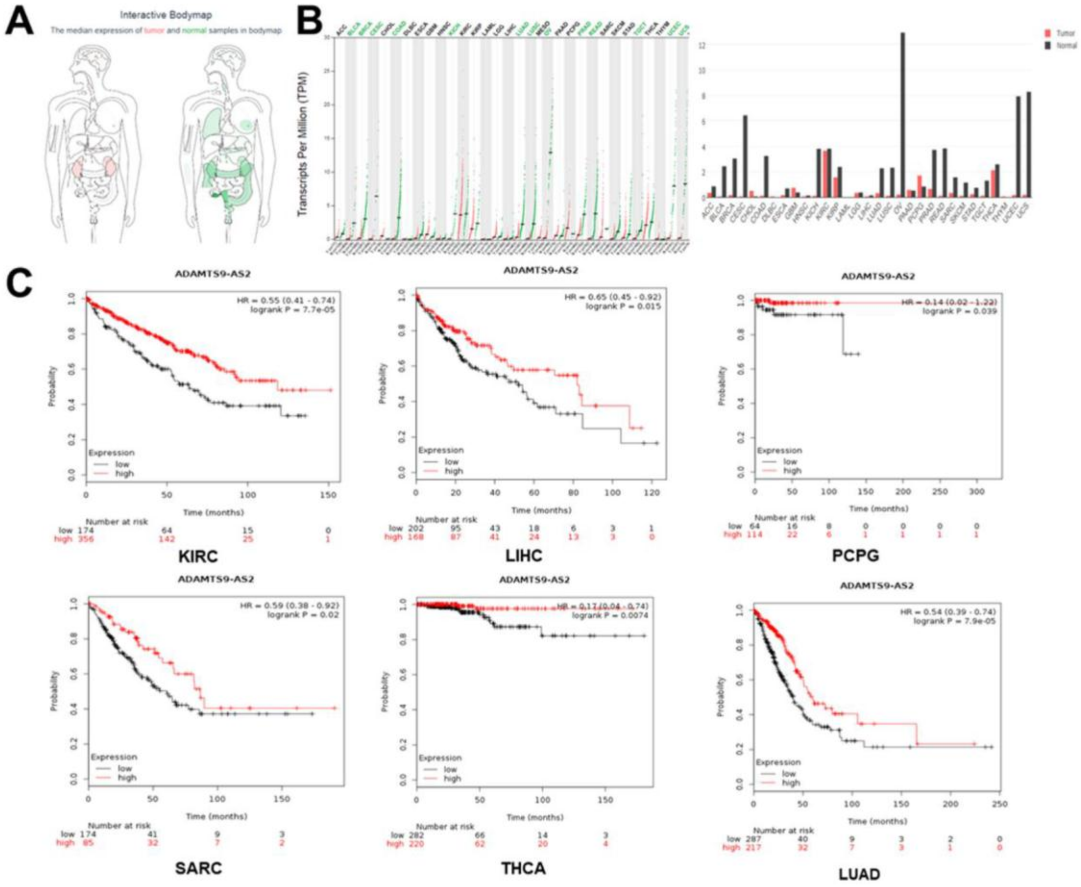

Supplementary Figure 2. The function of ADAMTS9-AS2 in the progression and development of various human cancers according to bioinformatics analyses. (**A**) The interactive bodymap of ADAMTS9-AS2 according to GEIPA database. Deeper color stands for higher level of ADAMTS9-AS2 expression. (**B**) The expression level of ADAMTS9-AS2 in various tumor tissues. (**C**) The association of ADAMTS9-AS2 expression with prognosis in six cancers according to KM plotter database. ADAMTS9-AS2, ADAM metallopeptidase with thrombospondin type 1 motif, 9 antisense RNA 2; GEIPA, Gene Expression Profiling Interactive Analysis; KIRC, kidney renal clear cell carcinoma; LIHC, liver hepatocellular carcinoma; PCPG, pheochromocytoma and paraganglioma; SARC, sarcoma; THCA, thyroid carcinoma; LUAD, lung adenocarcinoma; KM, Kaplan Meier.

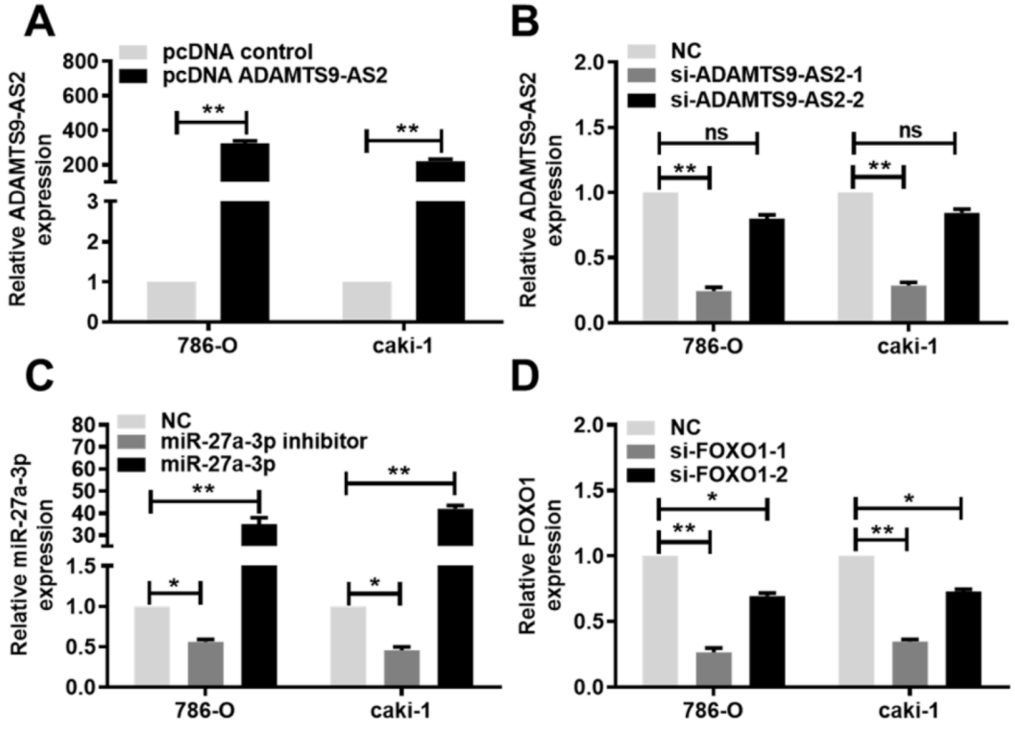

Supplementary Figure 3. The efficiency of overexpression and knockdown of ADAMTS9-AS2, miR-27a-3p and FOXO1 in 786-O and caki-1 cells. (**A**) Compared with pcDNA control, pcDNA ADAMTS9-AS2 significantly increased the expression levels of ADAMTS9-AS2 in 786-O and caki-1 cells. (**B**) Compared with NC, si-ADAMTS9-AS2-1 significantly decreased the expression levels of ADAMTS9-AS2, but si-ADAMTS9-AS2-2 showed no obvious effects on the expression levels of ADAMTS9-AS2 in 786-O and caki-1 cells. (**C**) Compared with NC, the expression levels of miR-27a-3p were significantly enhanced in miR-27a-3p group, whereas the expression levels of miR-27a-3p were evidently reduced in miR-27a-3p inhibitor group in 786-O and caki-1 cells. (**D**) Compared with NC, si-FOXO1-1 showed a higher inhibitory effect on FOXO1 expression than si-FOXO1-2 in 786-O and caki-1 cells. Three independent experiments were performed and data shown are mean ± SD. Statistically significant differences are indicated as *, $P<0.05$, **, $P<0.01$; ns, no significance; Student's *t*-test among two groups; ANOVA among multiple groups. ADAMTS9-AS2, ADAM metallopeptidase with thrombospondin type 1 motif, 9 antisense RNA 2; miR-27a-3p, microRNA-27a-3p; FOXO1, Forkhead Box Protein O1; NC, negative control; ns, no significance; SD, standard deviation.

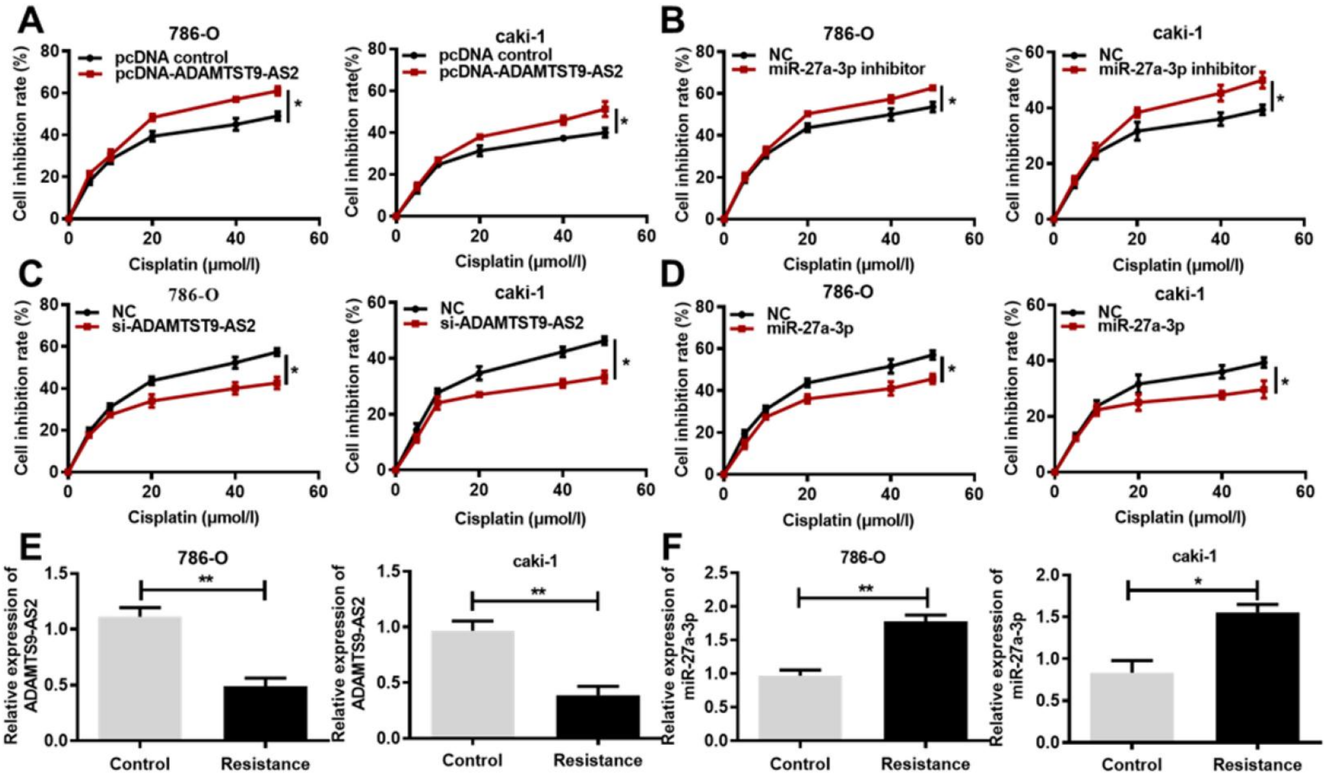

Supplementary Figure 4. ADAMTS9-AS2 overexpression and miR-27a-3p knockdown reduce ccRCC cell chemoresistance to Cisplatin. (**A**) MTS assays were performed in 786-O and caki-1 cells transfected with pcDNA ADAMTS9-AS2 or pcDNA control and treated with the indicated concentrations of Cisplatin. (**B**) MTS assays were performed in 786-O and caki-1 cells transfected with NC or miR-27a-3p inhibitor and treated with the indicated concentrations of Cisplatin. (**C**) MTS assays were performed in 786-O and caki-1 cells transfected with NC or si-ADAMTS9-AS2 and treated with the indicated concentrations of Cisplatin. (**D**) MTS assays performed in 786-O and caki-1 cells transfected with NC or miR-27a-3p and treated with the indicated concentrations of Cisplatin. (**E**) Expression levels of ADAMTS9-AS2 and (**F**) miR-27a-3p were determined by qRT-PCR in Cisplatin resistant 786-O and caki-1 cells. Three independent experiments were performed and data shown are mean ± SD. Statistically significant differences are indicated as *, $P<0.05$, **, $P<0.01$; Student's *t*-test. ADAMTS9-AS2, ADAM metallopeptidase with thrombospondin type 1 motif, 9 antisense RNA 2; miR-27a-3p, microRNA-27a-3p; ccRCC, clear cell renal cell carcinoma; si, small interfering; NC, negative control; MTS, 3-(4,5-dimethylthiazol-2-yl)-5-(3-carboxymethoxyphenyl)-2-(4-sulfop-henyl)-2H-tetrazolium); qRT-PCR, quantitative real-time polymerase chain reaction; SD, standard deviation.
