# Supplementary material for: LncRNA ADAMTS9-AS2 inhibits cell proliferation and decreases chemoresistance in clear cell renal cell carcinoma via the miR-27a-3p/FOXO1 axis
Source: Aging (Albany NY). 2019 Aug 10;11(15):5705–25. doi: 10.18632/aging.102154 (PMC6710069; doi:10.18632/aging.102154)
Supplement: Supplementary Table 1 [file aging-11-102154-s001.pdf]

SUPPLEMENTARY TABLE

Supplementary Table 1. List of oligoes used in this study.

Oligo name	Sequence 5′-3′
qPCR-ADAMTS9-AS2-F	AAGAAACCCTGATGTCTGGCTGAA
qPCR-ADAMTS9-AS2-R	GTGTTACTTGAGGAGAAAGCGAAA
qPCR-FOXO1-F	ATCTACGAGTGGATGGTCAA
qPCR-FOXO1-R	ATTGAGCATCCACCAAGAAC
qPCR-β-actin-F	CCACTGGCATCGTGATGGA
qPCR-β-actin-R	CGCTCGGTGAGGATCTTCAT
qPCR-miR-27a3p-F	TTCACAGTGGCTAAGTTCCGC
qPCR-miR-27a-3p-R	GCGAGCACAGAATTAATACGACTCACTATAGGTTTTTTTTTTTTTTTTTVN
qPCR-U6-F	CTCGCTTCGGCAGCACA
qPCR-U6-R	AACGCTTCACGAATTTGCGT
siRNA ADAMTS9-AS2-1	GCATGGGATGTATCTCCAT
siRNA ADAMTS9-AS2-2	CCTGTCTACAGGCTGATAT
siRNA FOXO1-1	GGAGAAGAGCTGCATCCAT
siRNA FOXO1-2	GAGGATTGAACCAGTATAT
miRNA-27a-3p mimic	UUCACAGUGGCUAAGUUCCGC
miRNA-27a-3p inhibitor	AAGUGTCACCGAUUCAAGGCG
pMS2ADAMTS9-AS2-F	CTAGCTAGCAAACTTGACGTACACACG
pMS2ADAMTS9-AS2-R	CGGGATCCTTTTCTGTTTTTATAATGTAC
pcDNA/pMS2ADAMTS9-AS2-MUT-F1	CTAGCTAGCAAACTTGACGTACACACG
pcDNA/pMS2ADAMTS9-AS2-MUT-R1	TGCCCCAGACACTGGGCTGCTGGGTCTTAAAGTTCCCA
pcDNA/pMS2ADAMTS9-AS2-MUT-F2	CTTGGGAACTTTAAGACCCAGCAGCCCAGTGTCTGGGGCA
pcDNA/pMS2ADAMTS9-AS2-MUT-R2	CGGGATCCTTTTCTGTTTTTATAATGTAC
psi-ADAMTS9-AS2-WT-F	AGCTTTGTTTAAACAAACTTGACGTACACACG
psi-ADAMTS9-AS2-WT-R	ATAAGAATGCGGCCGCTTTTCTGTTTTTATAATGTAC
psi-ADAMTS9-AS2-MUT-F1	AGCTTTGTTTAAACAAACTTGACGTACACACG
psi-ADAMTS9-AS2-MUT-R1	TGCCCCAGACACTGGGCTGCTGGGTCTTAAAGTTCCCA
psi-ADAMTS9-AS2-MUT-F2	CTTGGGAACTTTAAGACCCAGCAGCCCAGTGTCTGGGGCA
psi-ADAMTS9-AS2-MUT-R2	ATAAGAATGCGGCCGCTTTTCTGTTTTTATAATGTAC
psi-FOXO1 3′UTR-WT-F	CCGCTCGAGGGGTTAGTGAGCAGGTTACA
psi-FOXO1 3′UTR-WT-R	GGGTTTAAACAGGGCCTGAAACGTTGAATATGCA
psi-FOXO1 3′UTR-MUT-F1	CCGCTCGAGGGGTTAGTGAGCAGGTTACA
psi-FOXO1 3′UTR-MUT-R1	GACATGAGGCCCAATTACAAAAAGAGTATAAACTTTCCTTGGACC
psi-FOXO1 3′UTR-MUT-F2	GTTTATACTCTTTTTGTAATTGGGCCTCATGTCTTGATAAGT
psi-FOXO1 3′UTR-MUT-R2	GGGTTTAAACAGGGCCTGAAACGTTGAATATGCA

ADAMTS9-AS2, ADAM metallopeptidase with thrombospondin type 1 motif, 9 antisense RNA 2; qPCR, quantitative real-time polymerase chain reaction; F, forward; R, reverse; FOXO1, Forkhead Box Protein O1; si, small interfering; miRNA, microRNA; UTR, untranslated regions; WT, wild type; MUT, mutant type.
